# Psychophysiological correlates between emotional response inhibition and posttraumatic stress symptom clusters

**DOI:** 10.1038/s41598-018-35123-x

**Published:** 2018-11-15

**Authors:** Hongxia Duan, Li Wang, Jianhui Wu

**Affiliations:** 1 Center for Brain Disorder and Cognitive Science, Shenzhen Key Laboratory of Affective and Social Cognitive Science, Shenzhen University, Shenzhen, 518060 China; 2 Key Laboratory of Mental Health, Institute of Psychology, Chinese Academy of Sciences, Beijing, 100101 China

## Abstract

Post-traumatic Stress Disorder (PTSD) is characterized by diverse executive function impairments as well as abnormal emotion processing. The goal of the present study was to examine the relationships between emotional response inhibition and distinct PTSD symptom clusters from a six-factor DSM-5 model. Event-related potentials (ERPs) were measured in an emotional Go/NoGo task among 58 adult survivors from a deadly earthquake. Overall, the commission errors were lower and reaction time was faster for negative pictures compared to neutral pictures. The negative pictures elicited a smaller N2 but larger P3 amplitude compared to neutral and positive pictures, and larger P3 amplitude was further associated with a faster response. Multivariate regression models showed that the PCL score was related to smaller NoGo-N2 amplitude in the negative context, suggesting that the severity of posttraumatic stress symptoms is associated with worse conflict detection. Furthermore, the severity of anhedonia symptom cluster rather than negative affect symptom cluster was associated with fewer commission errors in the positive context, and this result provided electrophysiological evidence for the six-factor model, i.e., a distinction should be made between negative affect symptom cluster and anhedonia symptom cluster.

## Introduction

Posttraumatic stress disorder (PTSD) is a severe and complex mental disorder precipitated by exposure to a catastrophic event involving actual or threatened death or injury, or a threat to the physical integrity of him/herself or others (DSM-5)^1^. Impairments in cognitive functions, such as emotion, attention, memory and executive control functions are frequently observed in PTSD population^2–6^.

The capacity for continually monitoring and updating our actions is critical for effective performance in daily life. Response inhibition, the ability to inhibit prepared or proponent behaviors, is the key determinant of successful cognitive and motor control^7^. Neuroimaging studies have shown that fronto-basal ganglia networks, especially pre-supplementary motor area (preSMA), inferior frontal gyrus (IFG) and anterior cingulate cortex (ACC) are critical for inhibiting motor system and response tendencies^8–10^. Patients with PTSD generally exhibit abnormal top-down inhibitory control in the motor system, i.e., less activation in frontal brain areas and less deactivation of the motor cortex, suggesting there may be a deficiency of frontal inhibitory control to an enhanced motor readiness or an increased prepotency to respond^11–13^.

Studies with event-related brain potentials (ERPs), which have high time resolution, have also demonstrated abnormal inhibitory function at different processing stages in patients with PTSD. Specifically, during a Go/NoGo task, the frontocentral N2 elicited 200–400 ms following NoGo stimuli is considered an earlier step of response inhibition, i.e., detection of the conflict between the internal representation of the Go response and the NoGo stimulus^14–16^. The N2 is followed by a frontocentral P3 which is a positive component typically seen 300–700 ms post-stimulus onset, and this frontocentral P3 might represent a later stage of response inhibition, i.e., response evaluation/decision or response inhibition success^16,17^. Wu *et al*.^18^ found that adolescent earthquake survivors with PTSD exhibited a shorter NoGo-N2 latency than survivors without PTSD when performing a Go/NoGo task. Using a continuous performance task, Shucard *et al*.^19^ demonstrated that PTSD veterans had longer NoGo-P3 latency than civilian controls.

Incorporating emotionally salient stimuli into the Go/NoGo paradigm might help us elucidate the inhibition mechanism from the perspective of affective-modulated executive function. Previous studies showed that both implicit and explicit emotion stimulus processing modulates response inhibition^20–23^ and that behavioral dysregulation from psychiatric disorders became more prominent within emotional contexts^24^. From the perspective of PTSD, an exaggerated fear response and an inability to inhibit fear responses after trauma exposure are proposed to be a risk factor for the PTSD patients or an acquired trait of the illness^25^. The affective Go/NoGo task was proposed as a useful tool to assess psychiatric pathologies characterized by abnormal emotion processing^26^. Several studies with affective Go/NoGo paradigm in healthy participants have shown that emotional stimuli can influence the performance of response inhibition, as higher false alarm (incorrect NoGo response) rate for positive stimuli than negative and neutral stimuli^20,21,23,27^. With the method of ERPs, Zhang and Lu^28^ found smaller amplitudes and shorter latencies of Go-N2 following positive and negative faces than neutral faces, with larger P3 amplitudes and shorter P3 latencies for positive and negative faces than neutral faces in both Go and NoGo trials. On the other hand, Albert *et al*.^20^ found that NoGo-P3 amplitude is greater to positive pictures than to negative and neutral pictures. However, to our knowledge, there were no ERPs studies exploring the emotional response inhibition in individuals with posttraumatic stress symptoms to date.

PTSD is a highly heterogeneous clinical syndrome which is composed of distinct symptom clusters. Examining the relationships between cognitive functions, such as response inhibition, and severity of PTSD dimensional symptoms on a continuum rating may both yield higher reliabilities^29^ and help to further elucidate the psychopathology and core elements that underlie PTSD, in turn improving clinical assessment and intervention. A few studies to date have examined this relationship. For example, with three-factor DSM-IV model (i.e., re-experiencing, avoidance and hyperarousal)^30^, Swick *et al*.^31^ reported that commission errors were most strongly associated with re-experiencing symptoms in a behavior Go/NoGo task. Shucard *et al*.^19^ showed that NoGo-P3 latency was positively related to hyperarousal symptoms in Vietnam veterans. On the other hand, Wu *et al*.^32^ found that NoGo-P3 latency was positively associated with avoidance symptoms in a five-factor dysphoric arousal PTSD model (i.e., re-experiencing, avoidance, negative alterations in mood and cognitions, dysphoric arousal and anxious arousal) based on the DSM-5^1^.

In the most recent version of the DSM (DSM-5), negative affect and anhedonia are grouped into one cluster as negative alterations in mood and cognitions^1^. However, the cluster of negative alterations in mood and cognitions is a diverse construct within PTSD, which comprises symptoms involving enhanced negative affect/general distress and symptoms of reduced positive affect/anhedonia. Previous theoretical and empirical studies also suggested that negative affect and positive affect are different constructs^33–35^. More importantly, the Research Domain Criteria (RDoC) project initiated by the National Institutes of Mental Health (NIMH) specified that negative valence and positive valence are two distinct domains in psychopathology field^36,37^. Based on this evidence, Liu *et al*.^38^ proposed a new six-factor model in which the single symptom cluster of “negative alterations in mood and cognitions” from DSM-5 model was further divided into a negative affect symptom cluster (negative affect potentiation) and an anhedonia symptom cluster (positive affect deterioration) in an epidemiological sample of Chinese earthquake survivors. This six-factor model emerged as the best-fitting as compared to the current DSM-5 models^38^. Moreover, previous work from our own group showed that cortisol activity during cognitive task was only related with negative affect cluster, but not anhedonia cluster in participants who suffered from posttraumatic stress symptoms, providing preliminary evidence for this differentiation of negative affect and anhedonia^39^.

Thus, the aim of the present study was to explore the relationship between indices of emotional response inhibition (commission error and NoGo related ERP components) and each of the six symptom clusters from newly proposed PTSD six-factor model in trauma-exposed victims by using an emotional Go/NoGo paradigm adapted from Albert *et al*.^20^. To increase statistical power and decrease parameter estimation bias^40^, all participants, instead of only probable PTSD cases, were included in the regression analysis to capture the full range of symptom severity. This approach is also consistent with the RDoC proposal that the biological and clinical variables can be measured in a dimensional way, i.e., on a continuum ranging from normal to pathological^41,42^ as well as Lobo *et al*.^43^ suggestion that traumatized populations without PTSD diagnoses should also be included in dimensional analysis to identify putative EEG biomarkers of posttraumatic stress symptom severity. Based on the hallmark of this six-factor model and result from our previous work (distinction of negative affect and anhedonia), we predicted that the negative affect cluster and anhedonia cluster will have distinct associations with the behavioral performance or amplitude/latency of NoGo-N2/P3.

## Results

### Descriptive results

Table 1 showed the demographic and clinical variables of the study group (n = 58). The mean age of the participants was 50.16 ± 5.67 yrs. Among the 58 participants, 26 (44.83%) were male. The mean PCL-5 total score was 30.76 ± 16.06. The mean trauma exposure score was 5.12 ± 1.40, and the mean depression score was 41.16 ± 10.30.

**Table 1:** Demographic and clinical variables of the study group.

Variable	n	Mean	%	SDs	range
Sex					
*Male*	26		44.83		
*Female*	32		55.17		
Education Level					
*High school or above*	20		34.48		
*Less than high school*	38		65.52		
Age (yrs)		50.16		5.67	41–60
Trauma exposure		5.12		1.40	2–8
Depression		41.16		10.30	24–67
PCL-5		30.76		16.06	3–69
*RE*		9.78		4.43	1–20
*AV*		3.72		2.31	0–8
*NA*		4.74		3.93	0–14
*AN*		3.38		2.75	0–11
*DA*		5.95		3.84	1–16
*AA*		3.22		2.10	0–8

Note: PCL-5 = PTSD Checklist for DSM-5, RE = re-experiencing, AV = avoidance, NA = negative affect, AN = anhedonia, DA = dysphoric arousal, AA = anxious arousal.

### Behavioral performance

Table 2 showed the behavioral performance in the Go/NoGo task (means and standard deviations (SDs)).

**Table 2:** Descriptive statistics for the behavioral performance (n = 58).

	CE (%)			OE (%)			RT (ms)		
	Negative	Neutral	Positive	Negative	Neutral	Positive	Negative	Neutral	Positive
Mean	7.58	8.96	8.46	1.59	1.67	1.66	467.11	467.44	471.37
SDs	6.14	7.56	7.56	2.67	2.98	3.33	59.19	57.39	57.26

Note: CE = rate of commission errors in NoGo trials; OE = rate of omission errors in Go trials; RT = reaction time in the Go trials.

For the commission error (CE), there was a marginally significant valence effect (F(2,114) = 2.717, *p* = 0.070). Post-hoc analysis showed that participants have significantly lower commission error for negative than neutral pictures (*p* = 0.008). There was no significant difference between positive and negative pictures (*p* = 0.154) or between positive and neutral pictures (*p* = 0.464).

The grand mean of omission error (OE) rate was 1.6 ± 3.0%, and there was no valence effect on OE (F(2,114) = 0.133, *p* = 0.876).

For the RT of correct go trials, there was a significant valence effect (F(2,114) = 6.4053, *p* = 0.002). Post-hoc analysis showed that participants had a faster RT for negative/neutral than positive pictures (*p* = 0.002 and *p* = 0.008, respectively), but there was no significant difference between negative and neutral pictures (*p* = 0.781).

### ERPs

**Figure 1:**
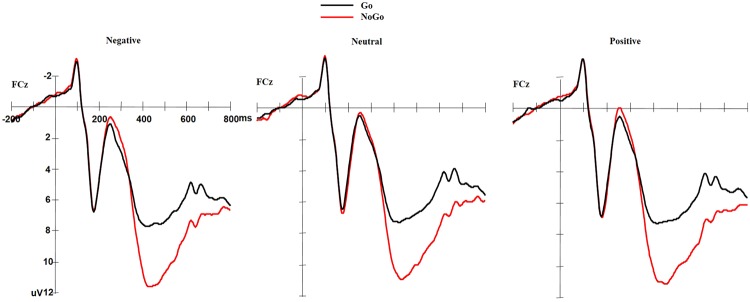
Grand average ERP for the NoGo and Go conditions at FCz under three different valence conditions.

As illustrated in Fig. 1, the N2 amplitude was larger for NoGo stimuli than for Go stimuli (F(1,57) = 8.843, *p* = 0.004). The valence main effect was also significant (F(2,114) = 6.233, *p* = 0.003), and post-hoc analysis showed that negative picture elicited a decreased N2 as compared to both neutral and positive pictures (*p* = 0.008 and *p* = 0.003, respectively), while there was no difference between neutral and positive pictures (*p* = 0.706). The interaction effect between valence and GoNoGo was not significant (F(2,114) = 1.42, *p* = 0.246).

For P3 amplitude, NoGo stimuli elicited significantly larger amplitude than Go stimuli (F(1,57) = 123.762, *p* = 0.000). The valence main effect was also significant (F(2,114) = 5.161, *p* = 0.007), and post-hoc analysis showed that negative picture elicited significantly larger P3 as compared to both neutral and positive pictures (*p* = 0.000 and *p* = 0.039, respectively), with no difference between neutral and positive pictures (*p* = 0.347). The interaction effect between valence and GoNoGo was not significant (F(2,114) = 0.292, *p* = 0.747).

There was no significant correlation between Go/NoGo-N2 amplitude and CE/RT in negative valence condition (*p*_*s*_ > 0.10). There was a significantly negative correlation between Go-P3 amplitude and RT (r = −0.351, *p* = 0.007) as well as between NoGo-P3 amplitude and RT (r = −0.381, *p* = 0.003) in negative valence condition, i.e., the larger of P3 amplitude, the faster participants responded to Go trials under negative context. There was no significant correlation between Go/NoGo-P3 amplitude and CE (*p*_*s*_ > 0.10).

### Regression analysis

#### The relationship between total PCL-5 and emotional response inhibition

**Figure 2:**
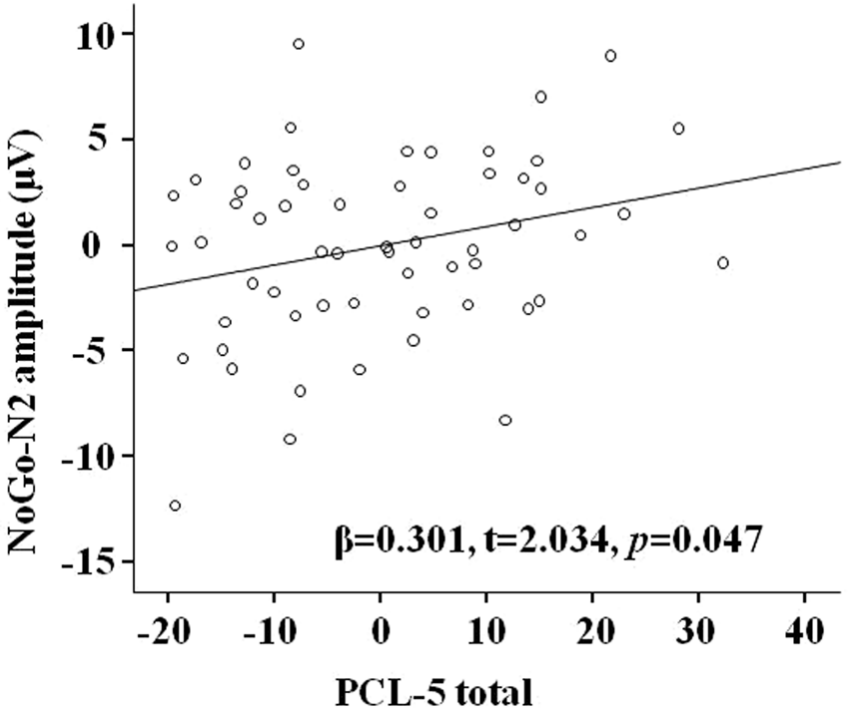
The partial regression scatter plot of NoGo-N2 amplitude under negative valence condition at FCz site with PCL-5 total score. Note: Because N2 is a negative component, thus the positive correlation coefficient, in fact, denotes a negative correlation, i.e., the higher of PCL-5 total score, the smaller of NoGo-N2 amplitude in the negative context.

The multivariate regression analyses showed that total PCL-5 score model predicted 27.3% for NoGo-N2 amplitude under negative valence condition (R^2^ = 0.273, F(6,57) = 3.197, *p* = 0.01): PCL-5 score had a positive association with NoGo-N2 amplitude (β = 0.301, t = 2.034, *p* = 0.047, see Fig. 2). Because N2 is a negative component, thus the positive correlation coefficient, in fact, denotes a negative correlation, i.e., the higher of total PCL-5 score the smaller of NoGo-N2 amplitude in negative context. There was no significant correlation between PCL-5 total score and NoGo-N2 amplitude in neutral or positive condition (*p*_*s*_ > 0.10).

No significant correlations were found between PCL-5 total score and commission error, NoGo-N2 latency, or NoGo-P3 amplitude/latency in each of the three valence conditions (*p*_*s*_ > 0.10).

#### The relationship between six symptom clusters and emotional response inhibition

**Figure 3:**
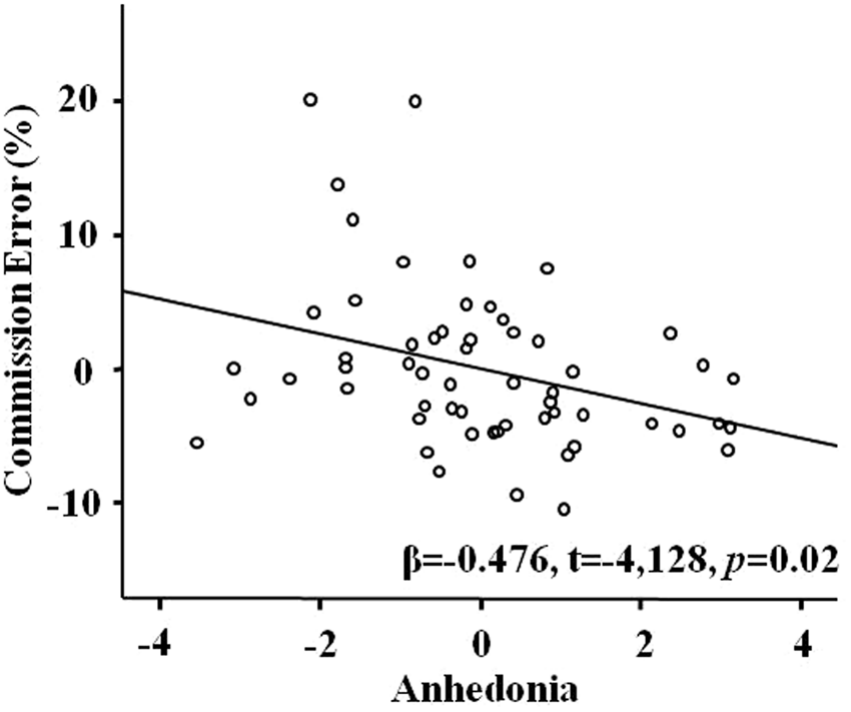
The partial regression scatter plot of commission error rate under positive valence condition with anhedonia score.

Multivariate regression analyses showed that the six-cluster model predicted 41.5% for commission error under positive valence condition (R^2^ = 0.415, F(11,57) = 2.972, *p* = 0.005): only anhedonia cluster had a negative association with it (β = −0.476, t = −2.418, *p* = 0.02, see Table 3 and Fig. 3). None of these six clusters were significantly associated with commission error under neutral or negative valence condition (*p*_*s*_ > 0.10).

**Table 3:** Results of regression analysis for whole participants with commission error under positive valence condition as a dependent variable and six clusters as independent variables (with age, gender, education, trauma exposure, and depression as covariates).

	β	t	*p*
RE	0.019	0.094	0.926
AV	−0.172	−0.857	0.396
NA	−0.053	−0.234	0.816
AN	−0.476	−2.418	0.020
DA	0.363	1.778	0.082
AA	0.084	0.416	0.679

Note: RE = re-experiencing, AV = avoidance, NA = negative affect, AN = anhedonia, DA = dysphoric arousal, AA = anxious arousal.

None of these six clusters models were significantly associated with NoGo-N2 amplitude/latency in each of three valence conditions (*p*_*s*_ > 0.10).

None of these six clusters models were significantly associated with NoGo-P3 amplitude/latency in each of three valence conditions (*p*_*s*_ > 0.10).

## Discussion

The current study investigated the relationship between posttraumatic stress symptom clusters based on the newly proposed six-factor PTSD model and response inhibition process under the emotional context at the behavioral and neural levels. First of all, the NoGo trials elicited larger N2 and P3 amplitudes than Go trials, which suggested the classical response inhibition effects on ERPs in this emotional Go/NoGo paradigm. Secondly, the results from the present study showed that emotional valence can modulate the response inhibition process: compared to in the neutral or positive context, participants committed fewer errors to NoGo and responded faster to Go in the negative context; there was a smaller N2 amplitude and larger P3 amplitude in the negative context compared to in the neutral or positive context. Furthermore, by utilizing a dimensional approach, we were able to identify distinct relationships between symptom clusters and distinct stages of response inhibition under emotional context within trauma-exposed individuals. We found that PCL-5 total score was negatively correlated with NoGo-N2 amplitude in the negative context, suggesting that PTSD severity is associated with worse conflict detection in the negative context. Furthermore, regression analysis showed that anhedonia, but not negative affect symptom cluster was associated with commission errors, which confirmed our hypothesis that anhedonia and negative affect symptoms should be differentiated.

Picture valence influenced the response inhibition performance. Behaviorally, participants had a lower commission error rate to negative pictures compared to neutral pictures. This result was partially consistent with several previous studies with healthy participants, as indicated by the fewer commission error for negative/neutral than positive stimuli (e.g., words, facial expressions, and pictures)^20,21,23,26,27^. These results suggested that “approach pleasant and reward-related events might make stopping responses to positive stimuli–more difficult than to other types of emotional stimuli”^20^, and avoidance of negative events makes stopping responses to negative stimuli easier in traumatized individuals.

On the neural level, ERP results showed that negative pictures elicited smaller N2 amplitude than neutral and positive pictures in traumatized individuals. The frontocentral N2 component has been proposed as an index of executive function, including attention deployment and conflict detection arising from competition between execution and inhibition of a single response in Go/NoGo task^10,53,54^, and the N2 amplitude increases with the amount of conflict and the extent to which cognitive control resources are recruited to detect current and future conflict^53,55^. In both normative and clinical populations, negative or threat-related stimuli can automatically capture attention and also interfere with the top-down cognitive control^56,57^. These negative interference effects have been explained by changes in processing priority that emotional processing is prioritized over cognitive processing when there is conflict or competition in the task^58,59^. Therefore, in the negative context, the facilitation of irrelevant negative characteristic in frequent Go trials (enhanced emotional engagement) may reduce the cognitive resources to detect the conflict between Go and NoGo trails. This study is among the first to show that N2 is sensitive to the emotional context in traumatized individuals. The reduced N2 amplitude to negative stimuli suggest poor conflict detection and less discrimination between Go and NoGo trials in the negative context.

The negative pictures elicited a larger P3 amplitude compared to neutral or positive stimuli, which was consistent with other studies in healthy population^60,61^. Furthermore, the correlation analysis showed that the larger P3 amplitude was associated with better behavior performance (RT to Go trials) in the negative context, which echoes the previous finding that larger P3 amplitudes in young adults were associated with more efficient working memory performance (RT and accuracy)^62^. P3 component reflects higher order cognitive processes such as evaluation of stimulus salience, categorization processing, and the subsequent memory encoding^63^. P3 amplitude increases with task relevance, motivational significance, arousal level, and top-down mental resource allocation^63^. Recent reviews proposed that P3 is modulated by dopaminergic activity which is exerted by the locus coeruleus-norepinephrine arousal system^64^. As stimuli with negative valence would be evaluated as more evolutionarily important, negative stimuli will elicit higher arousal level^56^ and recruit more top-down attentional resources^65^, thus leading to a larger P3 amplitude in the negative context. Therefore, the positive association between higher P3 amplitude and better performance under negative context suggested that individuals achieve better performance partially by recruiting more top-down attentional resources to the task.

Previous taxometric investigations have suggested that PTSD might be better classified as the upper extreme of the posttraumatic stress continuum instead of a categorical mental disorder in diagnosis and treatment^66–68^. In the present study, regression analysis within the whole group showed that PCL-5 total scores were negatively associated with NoGo-N2 amplitude only in the negative context. This result was consistent with the previous research that PTSD symptom severity is associated with worse inhibitory performance^11,69^. Our results further indicated that the overall PTSD symptoms severity was associated with the early stage of inhibition processing, i.e., poorer conflict detection, especially in the negative context.

Interestingly, we found that the severity of anhedonia symptoms was inversely related to commission error to NoGo trials only in the positive context, i.e., individuals with more anhedonia symptoms could hold their response better when response inhibition is needed in the positive context. Previous studies found that individuals with high level of anhedonia reported diminished positive experience to stimuli in a lab^70^ as well as in daily life situation^71^. Frewen and colleagues^72–74^ have found that participants with severe trauma exposure reported decreased positive emotion to positive stimuli, and this decreased positive response was related with anhedonia severity and increased amygdala activation. These findings indicated that less processing of positive stimuli or under-engagement of appetitive motivation system in individuals with high anhedonia level could generate less interference when inhibition response is needed, which might explain the association between anhedonia and the lower commission error to positive stimuli in the present study.

In the DSM-5, negative affect and anhedonia are grouped into one cluster as negative alterations in mood and cognitions^1^. However, within PTSD, negative alterations in mood and cognitions is a diverse construct involving symptoms of enhanced negative affect/general distress and symptoms of reduced positive affect/anhedonia. Furthermore, the RDoC project by the NIMH specified that negative valence and positive valence are two distinct domains in psychopathology field^36,37^. Previous theoretical and empirical studies also suggested that negative affect and positive affect are two different constructs^33–35^. This study showed that only anhedonia cluster, but not the negative affect cluster, was associated with commission error, which provides electrophysiological support for the current distinction of anhedonia and negative affect symptoms in the 6-factor DSM-5 model.

The present study had several limitations. First, we use a self-report measure, the PCL, to evaluate PTSD symptoms. The clinically administered instruments will be needed in the future studies. Secondly, all the participants were exposed to a deadly earthquake, thus the generalizability of the current findings was limited. Last but not the least, we only measured the response inhibition process among the survivors about five years after the trauma, thus it is hard to discriminate whether our results reflect the vulnerability factor before the trauma or consequences after the trauma. Nevertheless, the emotional response inhibition paradigm we took in this study is a promising first step towards the development of an electrophysiological index to explore the emotional inhibitory control in individuals with posttraumatic stress symptoms.

In conclusion, results from the present study showed that emotional stimuli can influence response inhibition. The negative stimuli impair conflict detection (smaller N2 amplitude) by prioritizing irrelevant emotional processing over cognitive processing on the one hand. On the other hand, negative stimuli facilitated behavioral performance (faster RT) partially by recruiting more top-down attentional resources to the task at hand (larger P3 amplitude). Consistent with prior work^32,66,75^, our study further emphasized the importance of assessing symptom severity dimensionally to improve our understanding of psychiatric disorders. The clinical–electrophysiological regression results showed that the severity of posttraumatic stress symptoms is associated with impaired inhibition processing at an early stage, i.e., poorer conflict detection in the negative context. Furthermore, the severity of anhedonia rather than negative affect symptom cluster was related with less commission error only in the positive context, which might be associated with less interference from positive emotion by under-engagement of motivational system. This result also provided electrophysiological evidence for the six-factor model, i.e., a distinction should be made between negative affect symptom cluster and anhedonia symptom cluster.

## Methods

### Participants

Participants were recruited through advertisements posted at local resident communities in Hanwang county. The participants selected were those who had been directly exposed to the devastating earthquake in Wenchuan County, Sichuan Province, China, on May 12, 2008, and with the current age range from 41 to 60 years. We excluded participants by self-report with (1) significant substance abuse, including drug, alcohol, and nicotine; (2) a past or current head injury; (3) self-reported neurological diseases or other serious medical condition. Sixty-one qualified volunteers meeting the inclusion and exclusion standards were selected to participate. The data from three participants were discarded because of too few accepted ERP trials due to poor behavioral performance and/or excessive movement artifacts.

All the participants didn’t take any psychiatric medication for at least four weeks before the experiment, which was conducted from 13–31 December 2013, approximately five and a half years after the earthquake. All the participants reported normal hearing and normal or corrected-to-normal vision. There were four participants reporting left-hand dominance.

### Questionnaires

The PTSD Checklist for DSM-5 (PCL-5) used in the current study is a self-report instrument to assess the PTSD symptoms^44,45^. The PCL-5 is adapted from the original PCL to map onto PTSD symptoms of the DSM-5^45^, and it includes six symptom clusters according to Liu *et al*.^38^: re-experience (B1–B5), avoidance (C1–C2), negative affect (D1–D4), anhedonia (D5–D7), dysphoric arousal (E1–E2, E5–E6), and anxious arousal (E3–E4). In the PCL-5, respondents rate all the 20 items from 0 (not at all) to 4 (extremely), to measure the severity of a particular symptom that has bothered them during the past month. The original PCL has been proven, reliable and valid^46,47^, and the Chinese version has been applied in previous research on earthquake-related PTSD symptomatology^38,40^.

The Center for Epidemiological Studies-Depression Scale (CESD) was used to assess depressive symptoms in the past week^48^. The version used here was translated into Chinese and its reliability and validity have been confirmed^49^.

The severity of trauma exposure was assessed by Trauma Exposure Scale^38^.

### Stimuli

Thirty trauma-unrelated pictures were selected from the International Affective Picture System (IAPS)^50^, including ten positive pictures, ten negative and ten neutral pictures. These pictures were re-rated by 48 Chinese volunteers, and analysis showed that positive > neutral > negative pictures at the valance value (7.29 ± 0.21 vs. 5.11 ± 0.25 vs. 2.88 ± 0.45, *p*_*s*_ < 0.001). Positive pictures have a similar arousal value as negative pictures (5.69 ± 0.65 vs. 5.63 ± 0.75, *p* > 0.05), and both positive and negative pictures have significantly higher arousal scores than neutral pictures (4.64 ± 1.14) (*p*_*s*_ < 0.05). Pictures were edited into three color frames, i.e., red, blue and green, resulting in 90 pictures total. All pictures were presented on a black background in the center of a computer screen at a visual angle of approximately 10° horizontally and 8° vertically.

### Procedure

After completing the questionnaires, the participants were seated comfortably in a normally lit room and completed an emotional Go/NoGo task which was adapted from Albert *et al*.^20^. After a practice block of 18 trials, two experimental blocks were completed with a short break between the blocks. Each block consisted of 45 pictures and each of them repeated four times, thus 180 trials for each block (1/3 NoGo and 2/3 Go). Each trial started with the presentation of a color framed picture for 300 ms, followed by a 1000–1400 ms interval with a white central fixation-cross presented on a black screen. The participants were required to either press a button (Go) as accurately and quickly as possible when pictures with one of the two color frames (e.g., red and blue: Go stimulus) were presented, or withhold their response (NoGo) when pictures with the other color frame (e.g., green: NoGo stimulus) were presented. Go/Nogo and picture valance conditions were presented in random order. The frame color indicating NoGo was counterbalanced across participants.

### EEG Recording and Preprocessing

An electroencephalogram (EEG) was recorded from 64 scalp sites using Ag/AgCl electrodes mounted in an elastic cap (Compumedics Neuroscan, Charlotte, NC). The EEG had an online reference to the left mastoid and an offline algebraic reference to the average of the left and right mastoids. The vertical and horizontal electrooculograms were recorded from two pairs of electrodes. One pair was placed above and below the left eye, and the other, 10 mm from the outer canthi of each eye. Interelectrode impedance was maintained at <5 kΩ. The signals were amplified with a 0.05–100 Hz bandpass filter and digitized at 500 Hz.

The EEG data were digitally filtered using a 30-Hz low-pass filter and were epoched into periods of 1000 ms (including a 200 ms prestimulus baseline) time-locked to the onset of the picture. Ocular artifacts were removed from the EEG signal using a regression procedure available through Neuroscan software. Trials with various artifacts were rejected if they exceeded the criterion of ±70 μV. The ERPs from both the Go and NoGo conditions were individually averaged. Behaviorally incorrect trials were not included in the ERP analysis.

The peak amplitudes and latencies of the frontocentral N2 and frontocentral P3 were measured at the Fz (frontal region), FCz (frontocentral region), and Cz (central region) sites. The N2 was defined as the minimum voltage in the time-window between 200 and 400 ms after stimulus onset, and P3 was defined as the maximum voltage in the time-window between 300 and 500 ms after stimulus onset. These sites and time windows were chosen in agreement with the previous literature^32,51,52^.

### Data Analysis

All statistical analyses were conducted using SPSS software (version 19.0). Descriptive statistics were gathered from the questionnaire scores and behavioral data, including the reaction time (RT) in correct trials, the rate of omission errors for the Go trials, and the commission error (CE) for the NoGo trials in each of all three valence picture conditions. Repeated measures analysis of variance (ANOVA) was carried out on behavioral performance and latency/amplitude at the three measurement sites (F_Z_, FCz, and Cz) under each of the three valence conditions (negative, neutral and positive). The Greenhouse–Geisser correction for degrees of freedom was applied when the sphericity assumption was violated. For cases where repeated measures ANOVA procedures revealed a significant main effect, post hoc analyses of least square difference were used to examine the specific effects and significant levels. To explore if there were relationships between behavior performance and electrophysiological index of response inhibition, the Person correlation coefficiency was calculated between N2/P3 amplitude and CE/RT.

Multivariate regression analyses were conducted to examine the associations between the six symptom clusters and indices of emotional response inhibition (behavioral performance and ERP components to NoGo condition). Amplitudes/latencies for the NoGo-N2 and NoGo-P3 components extracted at FCz site under each of the three valences (positive, neutral, and negative) were treated as dependent variables in regression analysis, as the amplitudes for both N2 and P3 were largest at this site. The symptom scores were treated as predictors, and demographic variables (age, gender, education level) and clinical variables (trauma exposure and depression) were entered as covariates in the regression analyses. The same method was also used to test the relationship between total PTSD severity and emotional response inhibition. All *p* values below 0.05 were considered statistically significant, and the tests were two-tailed.

### Ethics

All the participants gave written informed consent and were paid for their participation. The experiment was approved by the Ethics Committee of Human Experimentation at the Institute of Psychology, Chinese Academy of Sciences. The experiment was conducted in accordance with relevant guidelines and regulations.

## Data Availability

The anonymous behavioral and EEG data will be made available for research purposes upon requests.

## References

[1] American Psychiatric Association (APA). *Diagnostic and statistical manual of mental disorders (5th edition)*. (American Psychiatric Association, 2013).

[2] Ahmed F, Spottiswoode BS, Carey PD, Stein DJ, Seedat S (2012). Relationship between neurocognition and regional brain volumes in traumatized adolescents with and without posttraumatic stress disorder. Neuropsychobiology.

[3] Aupperle RL, Melrose AJ, Stein MB, Paulus MP (2012). Executive function and PTSD: disengaging from trauma. Neuropharmacology.

[4] Buckley TC, Blanchard EB, Neill WT (2000). Information processing and PTSD: a review of the empirical literature. Clin. Psychol. Rev..

[5] Carrion VG, Garrett A, Menon V, Weems CF, Reiss AL (2008). Posttraumatic stress symptoms and brain function during a response-inhibition task: an fMRI study in youth. Depress. Anxiety.

[6] Stein MB, Kennedy CM, Twamley EW (2002). Neuropsychological function in female victims of intimate partner violence with and without posttraumatic stress disorder. Biol. Psychiatry.

[7] Chambers CD, Garavan H, Bellgrove MA (2009). Insights into the neural basis of response inhibition from cognitive and clinical neuroscience. Neurosci. Biobehav. Rev..

[8] Aron AR, Robbins TW, Poldrack RA (2014). Inhibition and the right inferior frontal cortex: one decade on. Trends Cogn. Sci..

[9] Botvinick MM, Cohen JD, Carter CS (2004). Conflict monitoring and anterior cingulate cortex: an update. Trends Cogn. Sci..

[10] Huster RJ, Enriquez-Geppert S, Lavallee CF, Falkenstein M, Herrmann CS (2013). Electroencephalography of response inhibition tasks: functional networks and cognitive contributions. Int. J. Psychophysiol..

[11] Falconer E (2008). The neural networks of inhibitory control in posttraumatic stress disorder. J. Psychiatry Neurosci..

[12] Jovanovic T (2013). Reduced neural activation during an inhibition task is associated with impaired fear inhibition in a traumatized civilian sample. Cortex.

[13] van Rooij SJ (2014). Impaired right inferior frontal gyrus response to contextual cues in male veterans with PTSD during response inhibition. J. Psychiatry Neurosci..

[14] Donkers FC, Van Boxtel GJ (2004). The N2 in go/no-go tasks reflects conflict monitoring not response inhibition. Brain Cogn..

[15] Kok A (1986). Effects of degradation of visual stimuli on components of the event-related potential (ERP) in go/nogo reaction tasks. Biol. Psychol..

[16] Smith JL, Johnstone SJ, Barry RJ (2008). Movement-related potentials in the Go/NoGo task: the P3 reflects both cognitive and motor inhibition. Clin. Neurophysiol..

[17] Bruin KJ, Wijers AA, Van Staveren ASJ (2001). Response priming in a go/nogo task: do we have to explain the go/nogo N2 effect in terms of response activation instead of inhibition?. Clin. Neurophysiol..

[18] Wu J (2010). Response inhibition in adolescent earthquake survivors with and without posttraumatic stress disorder: a combined behavioral and ERP study. Neurosci. Let..

[19] Shucard JL, McCabe DC, Szymanski H (2008). An event-related potential study of attention deficits in posttraumatic stress disorder during auditory and visual Go/NoGo continuous performance tasks. Biol. Psychol..

[20] Albert J, López-Martín S, Tapia M, Montoya D, Carretié L (2012). The role of the anterior cingulate cortex in emotional response inhibition. Hum. Brain Mapp..

[21] Amin Z, Epperson CN, Constable RT, Canli T (2006). Effects of estrogen variation on neural correlates of emotional response inhibition. Neuroimage.

[22] Goldstein M (2007). Neural substrates of the interaction of emotional stimulus processing and motor inhibitory control: an emotional linguistic go/no-go fMRI study. Neuroimage.

[23] Schulz KP (2007). Does the emotional go/no-go task really measure behavioral inhibition? Convergence with measures on a non-emotional analog. Arch. Clin. Neuropsychol..

[24] Posner MI (2003). An approach to the psychobiology of personality disorders. Dev. Psychopath..

[25] Jovanovic T, Ressler KJ (2010). How the neurocircuitry and genetics of fear inhibition may inform our understanding of PTSD. Am. J. Psychiatry.

[26] Chiu PH, Holmes AJ, Pizzagalli DA (2008). Dissociable recruitment of rostral anterior cingulate and inferior frontal cortex in emotional response inhibition. Neuroimage.

[27] Putman P, van Peer J, Maimari I, van der Werff S (2010). EEG theta/beta ratio in relation to fear-modulated response-inhibition, attentional control, and affective traits. Biol. Psychol..

[28] Zhang W, Lu J (2012). Time course of automatic emotion regulation during a facial Go/Nogo task. Biol. Psychol..

[29] Brown TA, Campbell LA, Lehman CL, Grisham JR, Mancill RB (2001). Current and lifetime comorbidity of the DSM-IV anxiety and mood disorders in a large clinical sample. J. Abnorm. Psychol..

[30] American Psychiatric Association. Diagnostic and statistical manual of mental disorders (4th edition). (American Psychiatric Publishing, 1994).

[31] Swick D, Honzel N, Larsen J, Ashley V, Justus T (2012). Impaired response inhibition in veterans with post-traumatic stress disorder and mild traumatic brain injury. J. Int. Neuropsychol. Soc..

[32] Wu J (2015). The relationship between response inhibition and posttraumatic stress symptom clusters in adolescent earthquake survivors: an event-related potential study. Sci. Rep..

[33] Watson D (2005). Rethinking the mood and anxiety disorders: a quantitative hierarchical model for DSM-V. J. Abnorm. Psychol..

[34] Watson D (2009). Differentiating the mood and anxiety disorders: a quadripartite model. Annu. Rev. Clin. Psychol..

[35] Watson D, Clark LA, Stasik SM (2011). Emotions and the emotional disorders: a quantitative hierarchical perspective. Int. J. Clin. Health. Psychol..

[36] Cuthbert BN, Insel TR (2010). Toward new approaches to psychotic disorders: the NIMH Research Domain Criteria project. Schizophr. Bull..

[37] Cuthbert BN, Kozak MJ (2013). Constructing constructs for psychopathology: the NIMH research domain criteria. J. Abnorm. Psychol..

[38] Liu P (2014). The underlying dimensions of DSM-5 posttraumatic stress disorder symptoms in an epidemiological sample of Chinese earthquake survivors. J. Anxiety Disord..

[39] Duan H (2015). The relationship between cortisol activity during cognitive task and posttraumatic stress symptom clusters. PloS One.

[40] Wang L (2015). Assessing the underlying dimensionality of DSM-5 PTSD symptoms in Chinese adolescents surviving the 2008 Wenchuan earthquake. J. Anxiety Disord..

[41] Craske MG (2012). The R‐DoC initiative: Science and practice. Depress. Anxiety.

[42] Simmons JM, Quinn KJ (2013). The NIMH Research Domain Criteria (RDoC) Project: implications for genetics research. Mamm. Genome.

[43] Lobo I (2015). EEG correlates of the severity of posttraumatic stress symptoms: a systematic review of the dimensional PTSD literature. J. Affect. Disord..

[44] Blevins, C. A., Weathers, F. W., Witte, T. K., & Davis, M. T. The Posttraumatic Stress Disorder Checklist for DSM-5 (PCL-5): preliminary psychometric analysis in trauma-exposed college students. In Paper presented at the 28th annual meeting of the international society for traumatic stress studies. Los Angeles, CA (2012).

[45] Weathers, F. W., *et al* The PTSD checklist for DSM-5 (PCL-5). Scale available from the National Center for PTSD, http://www.ptsd.va.gov (2013).

[46] McDonald SD, Calhoun PS (2010). The diagnostic accuracy of the PTSD Checklist: a critical review. Clin. Psychol. Rev..

[47] Wilkins KC, Lang AJ, Norman SB (2011). Synthesis of the psychometric properties of the PTSD checklist (PCL) military, civilian, and specific versions. Depress. Anxiety.

[48] Radloff LS (1977). The CES-D Scale: a self-report depression scale for research in the general population. Appl. Psychol. Meas..

[49] Rankin SH, Galbraith ME, Johnson S (1993). Reliability and validity data for a Chinese translation of the Center for Epidemiological Studies-Depression. Psychol. Rep..

[50] Lang, P. J., Bradley, M. M., & Cuthbert, B. N. International affective picture system (IAPS): Affective ratings of pictures and instruction manual. *Technical report A-8 University of Florida*, *Gainesville*, *FL* (2008).

[51] Falkenstein M, Hoormann J, Hohnsbein J (1999). Erp components in go/nogo tasks and their relation to inhibition. Acta. Psychol..

[52] Sehlmeyer C (2010). Erp indices for response inhibition are related to anxiety-related personality traits. Neuropsychologia.

[53] Folstein JR, Van Petten C (2008). Influence of cognitive control and mismatch on the N2 component of the ERP: a review. Psychophysiology.

[54] Nieuwenhuis S, Yeung N, Van Den Wildenberg W, Ridderinkhof KR (2003). Electrophysiological correlates of anterior cingulate function in a go/no-go task: effects of response conflict and trial type frequency. Cogn. Affect. Behav. Neurosci..

[55] Forster SE, Carter CS, Cohen JD, Cho RY (2011). Parametric manipulation of the conflict signal and control-state adaptation. J. Cogn. Neurosci..

[56] Carretié L, Martín-Loeches M, Hinojosa JA, Mercado F (2001). Emotion and attention interaction studied through event-related potentials. J. Cogn. Neurosci..

[57] Simpson JR (2000). The emotional modulation of cognitive processing: an fMRI study. J. Cogn. Neurosci..

[58] Meinhardt J, Pekrun R (2003). Attentional resource allocation to emotional events: an erp study. Cogn. Emot..

[59] Wood J, Mathews A, Dalgleish T (2001). Anxiety and cognitive inhibition. Emotion.

[60] Huang YX, Luo YJ (2006). Temporal course of emotional negativity bias: an ERP study. Neurosci. Lett..

[61] Conroy MA, Polich J (2007). Affective valence and P300 when stimulus arousal level is controlled. Cogn. Emot..

[62] Saliasi E, Geerligs L, Lorist MM, Maurits NM (2013). The relationship between p3 amplitude and working memory performance differs in young and older adults. Plos One.

[63] Polich J (2007). Updating P300: an Integrative Theory of P3a and P3b. Clin. Neurophysiol..

[64] Nieuwenhuis S, Aston-Jones G, Cohen JD (2005). Decision making, the P3, and the locus coeruleus-norepinephrine system. Psychol. Bull..

[65] Vuilleumier P, Huang YM (2009). Emotional attention: Uncovering the mechanisms of affective biases in perception. Curr. Dir. Psychol. Sci..

[66] Broman-Fulks JJ (2006). Taxometric investigation of PTSD: data from two nationally representative samples. Behav. Ther..

[67] Broman-Fulks JJ (2009). The latent structure of posttraumatic stress disorder among adolescents. J. Trauma. Stress.

[68] Ruscio AM, Ruscio J, Keane TM (2002). The latent structure of posttraumatic stress disorder: a taxometric investigation of reactions to extreme stress. J. Abnorm. Psychol..

[69] Leskin LP, White PM (2007). Attentional networks reveal executive function deficits in posttraumatic stress disorder. Neuropsychology.

[70] Martin EA, Becker TM, Cicero DC, Docherty AR, Kerns JG (2011). Differential associations between schizotypy facets and emotion traits. Psychiatry Res..

[71] Brown LH, Silvia PJ, Myin-Germeys I, Kwapil TR (2007). When the need to belong goes wrong: the expression of social anhedonia and social anxiety in daily life. Psychol. Sci..

[72] Frewen PA (2010). Social emotions and emotional valence during imagery in women with PTSD: affective and neural correlates. Psychol. Trauma.

[73] Frewen PA, Dean JA, Lanius RA (2012). Assessment of anhedonia in psychological trauma: development of the Hedonic Deficit and Interference Scale. Eur. J. Psychotraumatol..

[74] Frewen PA, Dozois DJ, Lanius RA (2012). Assessment of anhedonia in psychological trauma: psychometric and neuroimaging perspectives. Eur. J. Psychotraumatol..

[75] Henderson SE (2014). The neural correlates of emotional face-processing in adolescent depression: a dimensional approach focusing on anhedonia and illness severity. Psychiatry Res..

